# A Parallel Algorithm Framework for Feature Extraction of EEG Signals on MPI

**DOI:** 10.1155/2020/9812019

**Published:** 2020-05-27

**Authors:** Qi Xiong, Xinman Zhang, Wen-Feng Wang, Yuhong Gu

**Affiliations:** ^1^School of Electronics and Information Engineering, Xi'an Jiaotong University, Xi'an 710000, China; ^2^International College, Hunan University of Arts and Sciences, Changde 415000, China; ^3^School of Electronic and Electrical Engineering, Shanghai Institute of Technology, Shanghai 200235, China; ^4^Shihezi Medical School, Shihezi 832000, China

## Abstract

In this paper, we present a parallel framework based on MPI for a large dataset to extract power spectrum features of EEG signals so as to improve the speed of brain signal processing. At present, the Welch method has been wildly used to estimate the power spectrum. However, the traditional Welch method takes a lot of time especially for the large dataset. In view of this, we added the MPI into the traditional Welch method and developed it into a reusable master-slave parallel framework. As long as the EEG data of any format are converted into the text file of a specified format, the power spectrum features can be extracted quickly by this parallel framework. In the proposed parallel framework, the EEG signals recorded by a channel are divided into *N* overlapping data segments. Then, the PSD of *N* segments are computed by some nodes in parallel. The results are collected and summarized by the master node. The final PSD results of each channel are saved in the text file, which can be read and analyzed by Microsoft Excel. This framework can be implemented not only on the clusters but also on the desktop computer. In the experiment, we deploy this framework on a desktop computer with a 4-core Intel CPU. It took only a few minutes to extract the power spectrum features from the 2.85 GB EEG dataset, seven times faster than using Python. This framework makes it easy for users, who do not have any parallel programming experience in constructing the parallel algorithms to extract the EEG power spectrum.

## 1. Introduction

**Figure 1:**
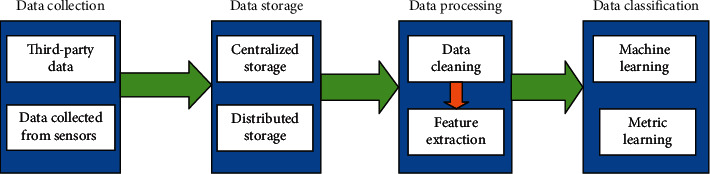
Steps of pattern recognition of the EEG signal.

EEG is a recorded signal of electrical activity of the brain, which is collected from the scalp through electrodes. The application of EEG has important practical value in medical treatment, military, sports, and the intelligence fields, which has been widely recognized by all the researchers. So far, scientists from various disciplines have achieved good results in this field. For instance, scientists from American Wadsworth Centre help the paralyzed people input 36 characters via signals instead of using their own fingers. These signals correspond to specific brain activity. In China, scientists from Tsinghua University have designed an automatic dialling system. The system is connected to the computer for real-time dialling by interpreting the thinking mode of the brain as corresponding numbers [1–6]. The research of pattern recognition of the EEG signal includes the following steps: data collection, data storage, data processing, data classification, and recognition as shown in Figure 1.

**Figure 2:**
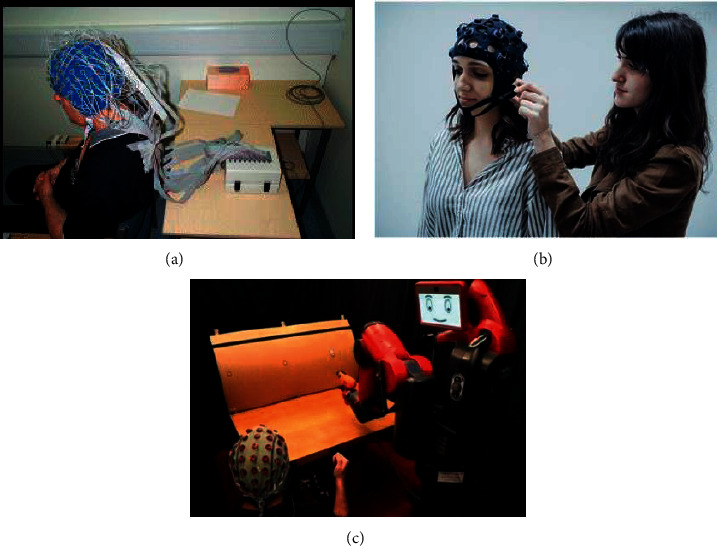
(a) 256-electrode device, (b) wearable EEG device, and (c) EEG device for disable.

In order to record EEG signals, several electrodes need to be placed on the scalp. Traditional device usually has 20 electrodes. However, recently, EEG devices with as many as 256 electrodes have been used, as shown in Figure 2(a). The increase in the number of electrodes enables recording of huge data thereby making the data processing stage in Figure 1 more important and complicated. This not only consumes a lot of computer resources but also leads to poor data extraction thereby directly affecting the accuracy of classification. There are many signal processing methods which can be used to extract EEG features with good discrimination. These methods include time domain analysis, frequency domain analysis, and time-frequency analysis. A comparative analysis of different approaches to spectral signal representation was performed in the paper [7]. These approaches include power spectral density (PSD) techniques, atomic decompositions, time-frequency (*t*-*f*) energy distributions, and continuous and discrete wavelet approaches, from which band power features can be extracted and used in the framework of motor imagery (MI) classification. It is pointed out that, among all the feature types of EEG signals, PSD approaches demonstrate to be the most consistent, robust, and effective in extracting the distinctive spectral patterns for accurate discrimination between left and right MI-induced EEGs.

At present, many methods are used to calculate the PSD of signals. The Welch method is one of the most popular, in which users calculate the PSD of the EEG signal in Python or Matlab environment. The function Scipy.signal.welch is used in Python and pwelch is used in Matlab. In case of small amount of data, PSD of the EEG signal can be quickly obtained by those two functions. But, from Figures 2(b) and 2(c), we can see that, with rapid developments in science and information technology and the upcoming of 5G era, wearable devices will widely be preferred. EEG signals of human activities can be collected by these devices. We can collect the current working or learning status of some specific people (such as drivers and students) via analysis of their EEG signals [8, 9]. If we calculate the PSD in the Matlab or Python environment, it will take a long time which we cannot bear it. Therefore, we focus on how to calculate the PSD of EEG signals by parallel approaches.

Parallel approaches can improve processing speeds but will need technologies that support distributed computations. Nowadays, there are two commonly different frameworks for big data analysis. One is Apache Spark, and the other is OpenMP/MPI. The two computational frameworks are compared and analyzed in literature [10]. Apache Spark has the advantage of good data management. OpenMP/MPI is faster than Apache Spark by an order of magnitude. Motivated by this analysis, we present a parallel framework for the large dataset to extract power spectrum features of EEG signals, which can be implemented in Linux and MPI environment.

**Figure 3:**
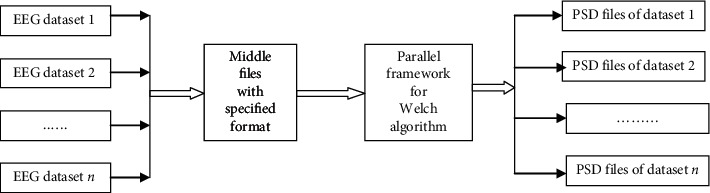
Relationship between the middle file and the parallel framework.

The main contributions of this paper are threefold:

1. According to the principle of Welch algorithm, we propose a parallel framework of Welch algorithm, PFwelch, to compute PSD of EEG. The architecture of PFwelch is based on the master-slave mode. The EEG signals recorded by each channel are divided into *N* overlapping data segments. Then, the *N* segments are computed by the master and slave node in parallel. The results are collected and summarized by the master node. The final Welch PSD results of each channel are saved in the text file, which can be read and analyzed by Microsoft Excel.
2. A middle file of the specified format was used. There are many kinds of EEG datasets in the world; different datasets have different file formats. In order to process any kind of EEG data by the proposed parallel framework, EEG data need to be converted into middle files. The relationship between the middle file and the parallel framework is depicted in Figure 3.
3. A comparative experiment was designed. In the experimental stage, we first run the function pwelch in the Matlab environment to extract PSD features from the EEG signal as a baseline for comparison and subsequently run PFwelch on the Ubuntu platform. The results show that the PFwelch have the same result as function pwelch. After this, we run the Python function Scipy.signal.welch in the same environment with PFwelch. The experimental result shows that the proposed parallel framework is 7 times faster than using Python.

This paper is organized as follows.  Section 2 takes a brief overview of the principle of the Welch method, and then presents a serial algorithm of the Welch method and the proposed parallel framework of the Welch method. In Section 3, we present experimental results and analysis. Discussion and conclusions are presented in Section 4.

## 2. Materials and Methods

### 2.1. Welch's Method

**Figure 4:**
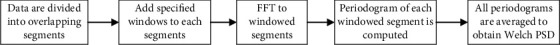
Welch PSD algorithm.

The power spectral density (PSD) exhibits how the power is contained in a signal in the frequency domain. Welch's method and the multitaper approach have shown the best performance among the PSD estimators [11]. The Welch algorithm [12] is exhibited in Figure 4.

From Figure 4, we can demonstrate the Welch algorithm in the following mathematical form.

The input signal *x*[*n*], *n*=0,1,…, *N* − 1 is split into many overlapping segments. In most cases, an overlap of 50% is applied when the input signal is divided into segments. Let the length of each segment be *L* and the total number of segments be *N*_*s*_. The formula for the data in the *i*th segment is as follows:

(1) $$\begin{array}{c} x_{i}=x\left[i\times \frac{L}{2}+n\right]\text{,}\;\text{where }n=\text{0},\ldots ,L-1\text{, }i=0,1,2,\ldots ,N-1. \end{array}$$

**Figure 5:**
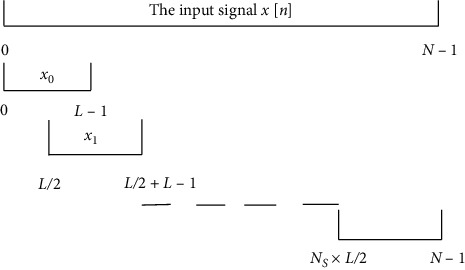
Illustration of signal segmentation.

The procedure of the segmentation is illustrated in Figure 5.

The relationship of the sampling length *N*, number of overlapping points *N*_D_, number of segments *N*_S_, and segmental length *L* is

(2) $$\begin{array}{c} N=L+\left(L-N_{D}\right)\left(N_{s}-1\right). \end{array}$$

**Figure 6:**
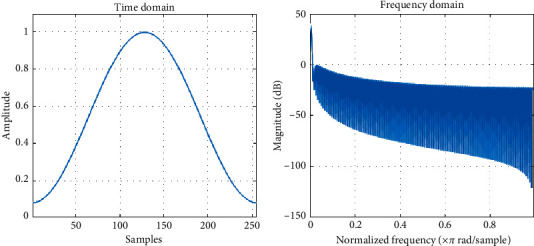
Hamming window in the time and frequency domain with Matlab.

A smooth window *w*(*n*) is applied to each segment. Generally, we usually use the Hamming window. The formula of the Hamming window for each segment is as follows:

(3) $$\begin{array}{c} w\left(n\right)=0.54-0.46\;\cos\left[\frac{2n\pi }{L}\right], \end{array}$$

where *n*=0,1,2 …, *L* − 1, *L* denotes the length of each segment.  Figure 6 displays a 256-point Hamming window in the time domain and frequency domain with Matlab.

**Figure 7:**
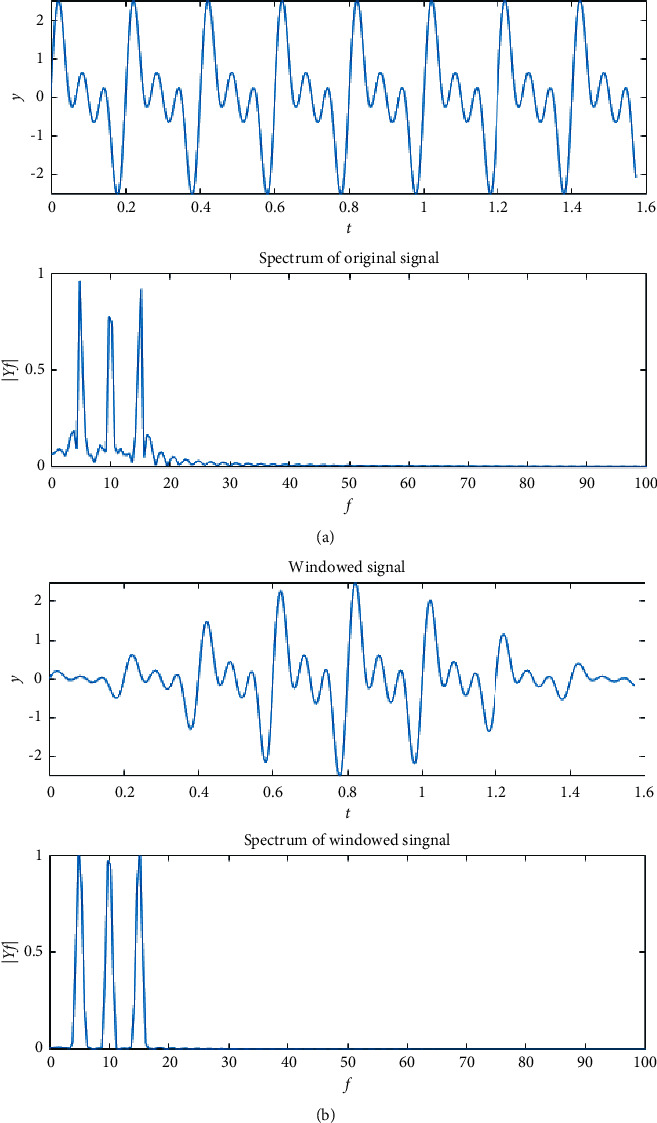
(a) The spectrum leakage of the original signal and (b) the windowed signal.

The purpose of the window function is to prevent the spectral leakage [13].  Figure 7(a) shows the spectrum leakage of the original signal.  Figure 7(b) exhibits that the spectrum leakage can effectively be reduced by the Hamming window.

From formula (3), we can get formula (4) for the *i*th segment of data after being windowed:

(4) $$\begin{array}{c} W_{i}=x_{i}\left(n\right)\times w\left(n\right). \end{array}$$

Fourier transform of each windowed segment is computed. The formula is as follows:

(5) $$\begin{array}{c} A_{i}\left(k\right)=x_{i}\left(n\right)w\left(n\right)e^{-\left(j\left(\left(2\pi \right)/N\right)nk\right)}, \end{array}$$

where *A*_*i*_ is the Fourier transform result of the *i*th windowed segment, *i*=0, 1,…, *L* − 1.

The periodogram of each windowed segment is computed by using the following formula:

(6) $$\begin{array}{c} \phi_{i}=\frac{1}{LU}{\left|A_{i}\left(k\right)\right|}^{2}, \end{array}$$

where *U*=(1/*L*)∑_*n*=0_^*L*−1^*w*^2^(*n*) denotes the mean power of the window *w*(*n*).

So, *LU*=∑_*n*=0_^*L*−1^*w*^2^(*n*) denotes the energy of the window function *w*(*n*) with length *L*.

Finally, we can get the PSD by the Welch method which is the average of those periodograms, i.e.,

(7) $$\begin{array}{c} S\left(k\right)=\frac{1}{L}\sum_{i=0}^{L-1}\phi_{i}\left(k\right). \end{array}$$

### 2.2. Serial Algorithm of the Welch Method

In order to design a good parallel program, it is necessary to understand the traditional serial algorithm. According to the above description of the Welch method, the serial algorithm of the Welch method is given as Algorithm 1.

### 2.3. Proposed Parallel Framework of the Welch Method

#### 2.3.1. Program Structure

**Figure 8:**
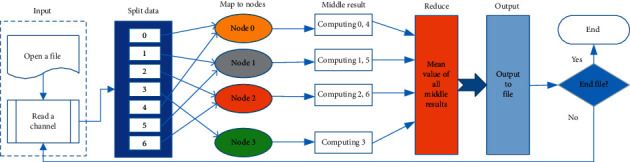
Program structure of PFWelch.

According to the steps of serial algorithms of the Welch method in Section 2.2, it can be seen that this algorithm can be implemented in parallel with MPI. The structure of the parallel algorithm is master-slave and is demonstrated as Figure 8.

The structure of PFWelch algorithm in Section 2.2 contains the following stages: input, split, map, reduce, and output stage. One channel data are split into seven segments, which are allocated to four nodes according to certain rules. All nodes are responsible for computing. In the reduce stage, the master process is responsible for receiving the result from slave processes and compute the final PSD. Since the parallel implementation of fast Fourier transform has been maturely developed [14–20], we need not give the relevant details. The parallel algorithm of the Welch method is described in Algorithm 2.

#### 2.3.2. Distribution of Tasks

The key point of the parallel program is how to cooperate with each node [21]. How to distribute tasks evenly to each computing node is one of the major factors affecting the performance of the parallel program. From Figure 8, the EEG data of each channel are divided into 7 segments (*n*_segs = 7). The length of each segment is 64. The allocation scheme is shown in Table 1 when the number of nodes is 4 (size = 4).

According to Table 1, it is convenient to calculate the signal range that each processor needs to compute. For example, processor 0 needs to handle segments 0 and 4. From formula (1), it can be calculated that the signal range is [0, 63] and [128, 191], respectively.

## 3. Experimental Results and Analysis

### 3.1. Dataset

In the experiment, the data were downloaded from the website http://kdd.ics.uci.edu/databases/eeg/eeg.html. This dataset arises from a large study to examine EEG correlates of genetic predisposition to alcoholism. It includes measurements from 64 electrodes placed on the scalp sampled at 256 Hz. There are three versions of the EEG data set: small data set, large data set, and full data set. The full data set contains all 120 trials for 122 subjects. The entire set of data is compressed to about 700 MB [22]. When it was uncompressed, its size is approximately 2.8 GB. After all files were converted into middle files, there were in all 11,058 files.

### 3.2. Testing Method and Environment

There are three different performances which need to be tested:

1. *Accuracy*. The result of MATLAB is compared with that of the parallel framework.
2. *Speed*. The running time of Python program is compared with that of the parallel framework.
3. *Speedup*. The parallel framework was run with different nodes. The running time was recorded, and speedup can be obtained.

The testing environment of hardware is shown in Table 2, and the testing environment of software is shown in Table 3.

### 3.3. Experimental Results

**Figure 9:**
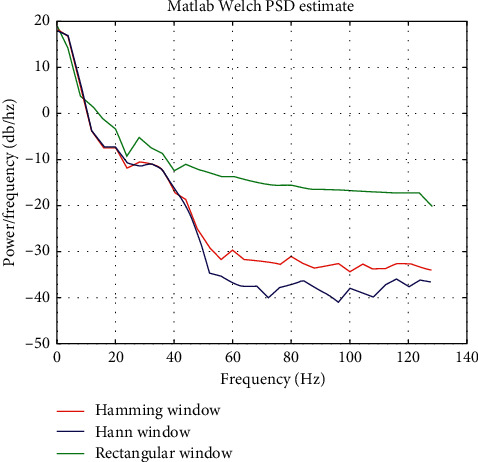
Welch result in Matlab.

PSD computation of one EEG channel was conducted in the Matlab by using the Welch function, i.e., pwelch (data, hamming(64), 32, 64, 256). The pwelch parameters mean that sample data are split up into segments, each segment has 64 data points, the overlap of neighboring segments is 32, Hamming window is applied, and the sampling rate is 256 Hz.  Figure 9 shows the result of the Welch method in three different window functions in Matlab.

**Figure 10:**
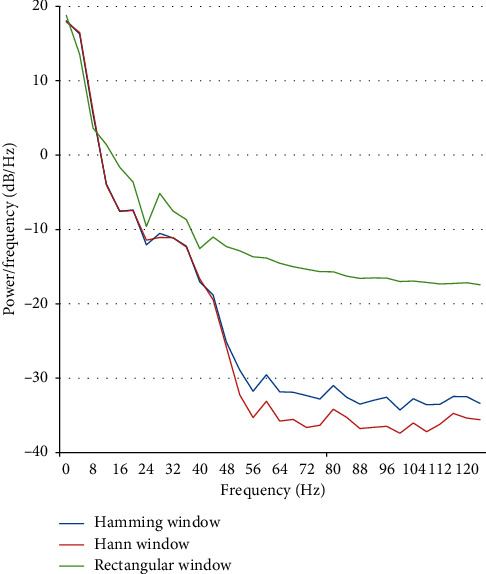
Result of PFWelch.

We performed the same test in the parallel framework. The PSD results were stored in the text file which can be accessed by Microsoft Excel and is shown in Figure 10.

Comparison between Figures 9 and 10 indicates that the results obtained by both Matlab and PFWelch are consistent, which corroborates the correctness of PFWelch. To test the time performance, we first used different number of nodes in different segments to calculate the PSD of one EEG file with PFWelch (in this experiment, we regard different CPU cores as different nodes). Time consumption (measured in seconds) is recorded in Table 4. For comparison, the time cost in Python environment by the serial algorithm is also listed in Table 4. As we have not used Matlab Linux version, we carried out such comparative experiments with Python.

Second, we use different number of nodes with 7 segments to calculate the PSD of all EEG files with PFWelch. The time cost is recorded in Table 5.

**Figure 11:**
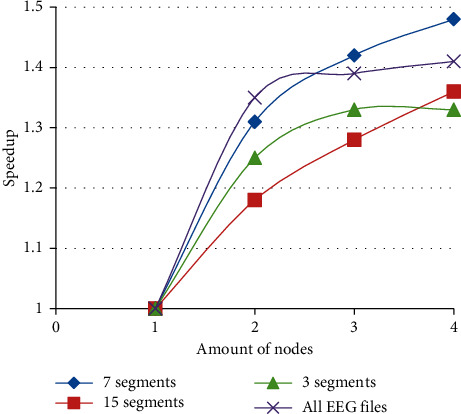
Speedup of PFWelch.

Speedup can evaluate the time performance of PFWelch. The definition of speedup is speedup = Ts/Tp, where Ts indicates the time of serial operations and Tp indicates the time of parallel operations. The speedups are shown in Figure 11. Figure 11 demonstrates that, as the number of nodes increases, the speedup also increases. But, in different cases, the increase is not the same. For all EEG files, Figure 11 shows that, as the number of nodes increases to a certain value, the speedup slowly increases. The reason is the time it takes includes files open and close. This time cannot be changed when the number of nodes is increased.

**Figure 12:**
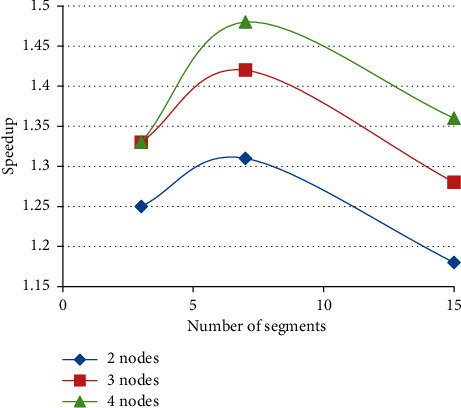
Relationship between the number of segments and speedup.

For a single EEG file, the PFWelch shows the best performance when the signal is split into 7 segments. This is because it has the maximum speedup when the number of nodes is 4. Therefore, we divided the signal into 7 segments to calculate the PSD of all EEG files in Figure 11. If the signal is split into 15 segments, the speedup grows basically in a linear manner, but its speedup is the lowest.  Figure 12 clearly reveals the relationship between the number of segments and the speedup.

It can be seen that, by increasing the number of nodes, the speedup is improved. But, merely increasing the number of nodes cannot improve the speedup. From Figure 12, we can see that the speedup decreases when the number of segments increases to 15. When the number of segments is 7 and the number of nodes is 4, we get the best speedup performance.

## 4. Discussion and Conclusion

At present, although there are several methods which can extract features from the EEG signal, the PSD is still one of the most important methods. But, while dealing with a large number of EEG data, it is necessary to improve the calculation speed of PSD. Therefore, the parallel framework proposed in this paper is used to solve the problem of taking long time to extract PSD features from the EEG dataset in big data environment. The framework is based on C + MPI language and is completed by the master-slave mode. Compared with the traditional serial Welch method, this framework divides the signal into *N* segments and distributes them evenly to different nodes on which PSD can be calculated in parallel. In the experiment, the values of *N* are 3, 7, and 15, respectively. The number of nodes is from 1 to 4. For the given data set, we found that, although the speedup can be improved by increasing the nodes, the speedup performance will decrease if there are too many segments. Experiments show that the best performance of the parallel framework can be achieved only when the number of nodes and segments is reasonably selected. The speed of PFWelch is 7 times faster than using Python in the same hardware and operating system platform.

Because of the powerful function of MPI, this framework can be deployed not only on the cluster but also on the desktop computer, which is very convenient for the users. The experimental results also corroborate that the framework is correct, efficient, and has a good practical value. It can be applied to extract all kinds of EEG datasets with a little modification. Researchers who are interested in PFWelch can download the source code from https://github.com/abcxq.

## Figures and Tables

**Algorithm 1:**
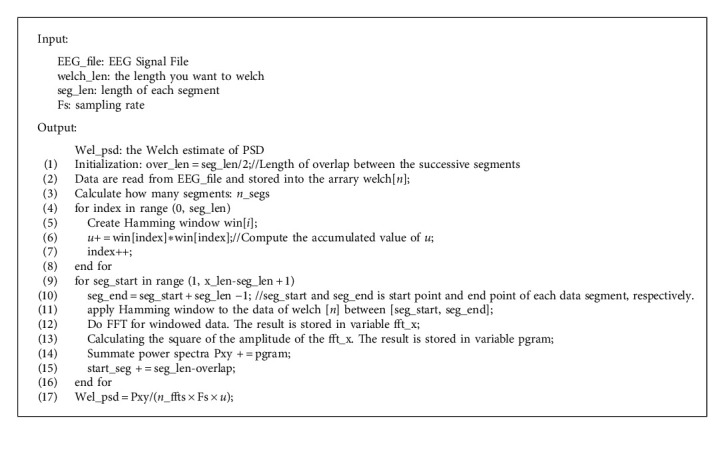
Serial algorithm of the Welch method.

**Algorithm 2:**
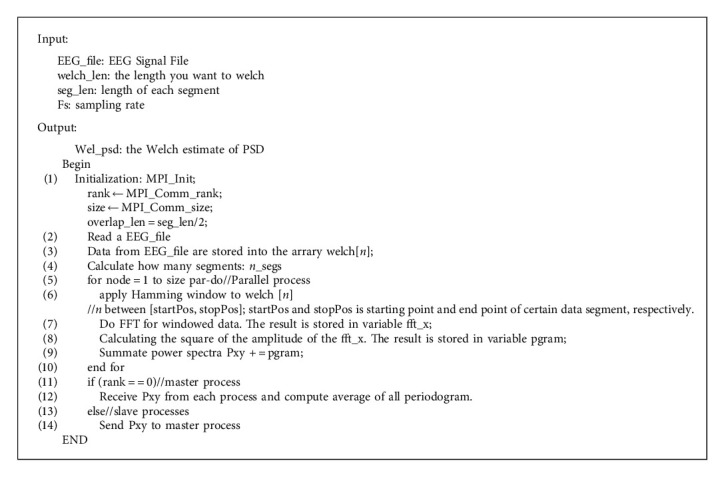
Parallel algorithm of the Welch method.

**Table 1:** Task allocation.

Label of the segment	0	1	2	3	4	5	6

Label of nodes	0	1	2	3	0	1	2

**Table 2:** Testing environment of hardware.

Nodes	CPU	Memory	Type
4	Intel(R) core(TM) i5-4460	8 GB	Desktop computer

**Table 3:** Testing environment of software.

Operating system	Parallel environment	Matlab
Ubuntu 16.04	C + Mpich 3.3.1 on Ubuntu	R2018a on Win10

**Table 4:** Time cost of one EEG file.

Segments	Nodes				
	1	2	3	4	Python
7	0.0543	0.0413	0.0382	0.0365	0.228
3	0.075	0.06	0.056	0.056	0.235
15	0.064	0.054	0.05	0.047	0.224

**Table 5:** Time cost of all EEG files.

Nodes	1	2	3	4	Python

Time cost (s)	478.778	354.008	342.715	337.966	2451

## Data Availability

All the data utilized in our research can be accessed from http://kdd.ics.uci.edu/databases/eeg/eeg.html.
